# A Proposed Model for Infant and Child Oral Health Promotion in India

**DOI:** 10.1155/2013/685049

**Published:** 2013-10-30

**Authors:** Ashwin Muralidhar Jawdekar

**Affiliations:** Department of Pediatric Dentistry, YMT Dental College and Hospital, Kharghar, Navi Mumbai 410210, India

## Abstract

Dental caries is an increasing burden in the developing countries. A proper budgetary allocation for treating dental diseases in an enormous population such as India is impractical, where resources are inadequate for major health challenges such as malnutrition and gastrointestinal and respiratory infections in children. An integrated, directed population approach targeting children is much needed. The existing machinery of successful public health campaigns such as the “Pulse Polio” and the “Mid-Day-Meals Scheme” of the Government of India can be used for oral health promotion for children. India has about 300 dental colleges and countrywide branches of the Indian Dental Association that can provide manpower for the program. An innovative, large-scale “Fit for School” program in Philippines is a model for an integrated approach for children's health and has proved to be cost-effective and viable. A model for oral health promotion in infants and children of India, combining age-specific initiatives for health education, nutrition, hygiene, and fluoride use, is proposed. The model could be implemented to evaluate the oral health status of children, knowledge and knowledge gain of the community health workers, and acceptability and sustainability of the preventive programs (fluoride varnish and preschool and school tooth brushing) pragmatically.

## 1. Introduction

Oral diseases are the commonest chronic diseases and are amongst the most expensive diseases to treat [1, 2]. Although oral health can be regarded as a fundamental human right, inequalities in oral health continue to exist globally [1]. Rich countries witnessed a marked reduction in the experience of dental caries in children and young adults during 1970 and 2000 [3]; however, in the developing countries, owing to the westernized diets and the consumption of sugar, dental caries increased during the same period [4].

The effect of untreated caries on the growth and well-being of children often remains ignored [5]. Children having poor oral conditions are three times more likely to remain absent in schools due to dental pain and also perform poorly in studies [6]. Oral diseases affect the quality of life of children and account for pain, impaired aesthetics, recurrent infections, eating troubles, sleeping difficulties, emergency visits to dentists and hospitals, poor ability to learn, insufficient nutrition, and improper growth and development [2]. Dental caries affects children socially as well as psychologically. Furthermore, treating dental caries in children is expensive due not only to the direct costs of treatment but also the indirect costs such as the time taken off by the parents to take the child to a dentist [5].

In the developing countries, millions of children die every year of preventable diseases as the scarcity of resources deprives them a basic package of preventive healthcare. Therefore, restoring decayed teeth remains well out of the reach of these countries due to the budgetary constraints, which means that more than 90% of the caries remains untreated. This calls for an integrated preventive approach for oral and general health [7]. Dental health is an integral aspect of general health. Oral diseases and other chronic diseases have “common risk factors” [1, 8]. In order to improve the population dental health effectively, a broadly based health promotion strategy should be devised based on the common risk factor approach [3].

## 2. The Population: India

India is the second most populous country in the world [9]. The technological and economic growth over the past few decades in India has been phenomenal [10]. However, India ranks low in the Human Development Index (134th among 182 countries in the year 2009) due to inadequate investment in health and education and poor living standards [11]. According to the government estimates, 29 percent people in India are below poverty line; moreover, a more sensitive index such as the *Multidimensional Poverty Index* (*MPI*) measures more than 55% of the Indians as poor [10]. In India, the private sector is responsible for the majority (71.6%) of the healthcare while the public sector accounts for 26.7% and external funding constitutes 1.7% [12]. Like many other developing countries, India has a huge burden of chronic noncommunicable diseases in addition to the continuing challenge of infectious diseases [11]. A high infant and child mortality rate (14.5% are infant deaths <1 years, 3.9% are deaths of 1–4 years children, 18.4% are deaths of children of 0–4 years, and 2.7% deaths of 5–14 years) and staggering high malnutrition (48%) are reported in a recent publication of the Central Statistics Office Ministry of statistics and Program Implementation, Government of India [13].

National Oral Health Survey and Fluoride Mapping was a comprehensive epidemiological investigation carried out by the Ministry of Health and Family Welfare, Central Government of India in the years 2002-2003 [14]. Important summary findings of the same are as follows.

There was a high caries experience across all age groups in India, with a high proportion of untreated decayed teeth (d/D) in children. The presence of the filled (f/F) component was negligible. In the 5-, 12-, and 15-year-old children, the prevalence of dental caries was 51.9%, 53.8%, and 63.1%, respectively [14, 15].

The report further stated that there was a skewed distribution of dental caries and the Significant Caries (SiC) Index was two or more times higher than dmft/DMFT levels. The mean dmft/DMFT scores for ages 5-, 12-, and 15-year-old children were 2.0, 1.8, and 2.4, respectively, and the corresponding SiC scores were 5.5, 3.0, and 4.1. There were no significant differences on the basis of gender. The population in rural India experienced more caries. The report also indicated a need for early treatment and prevention of dental caries in the population as the disease levels increased with advancing age [14].

## 3. Considerations for Developing a Model for Infant and Child Oral Health Promotion in Rural India

Most chronic diseases and oral conditions stem from the same risk factors which need to be controlled [8]. The challenge of infectious diseases in India is formidable and the causes of infections are poorly understood by people such as the open field defecation, inadequate sanitation (leading to contamination of drinking water), and poor hygiene practices [11]. The two strategies adopted by the government, the “selective disease control,” and “the ad-hoc outbreak control” often prove inadequate to limit the infectious diseases, particularly due to the lack of modern public health approaches of prevention [11]. The burden of noncommunicable diseases in India is enormous and the chronic diseases account for 53% of total deaths [16]. Diarrhoea, pneumonia, and insufficient nutrition continue to affect the health of children adversely [17]. 

Poor nutrition can be a cause as well as an effect of poor oral health [5]. The Western World has benefitted from the widespread use of fluoride dentifrices and population approaches such as water fluoridation that has resulted in decline in dental caries [3]. The current caries trend in India demands a robust prevention policy for the control of dental caries [14]. A “directed population” approach based on geographic targeting (rural areas in each district) and targeting on the basis of schools (government run and aided) in these rural areas can be the taken up to devise a strategy for oral health promotion.

It is needless to reemphasize that an ideal model for the oral health promotion in rural India should be cost effective, acceptable, sustainable, and aiming at overall health promotion. Moreover, a preventive program targeting children in deprived communities integrating oral health with general health (for the prevention of diarrhoea and respiratory infections and improving nutrition) has to be evidence based.

India adopted a primary healthcare approach as a result of the Alma Ata Declaration in 1978. There exists a wide network of primary health care centers and community health workers in rural India. In the recent past a few health promotion policies of the Government of India have been widely publicized and have also led to significant developments in the health scenario. These policies can be studied and regarded as the foundation for the development of an oral health promotion model. Two national public health programs are discussed in Sections 4 and 5.

## 4. “Mid-Day-Meals Scheme”

The “Mid-Day-Meals Scheme” was launched in India on 15th August (the day celebrated as the Independence Day) in the year 1995 by the Central Government of India. In a declaration, the government mandated free cooked meals made of 100 grams of cooked wheat or rice to all children in public schools across all states. The Supreme Court of India directed all the schools that had not implemented the program to do so within six months in the year 2001 [18]. The program is based on the fundamental “right to food” and continues to be the largest nutritional program in the world. The program saw a few significant revisions in the years 2004 and 2007 such as the children of upper primary schools (grade VIII-X) and providing additional nutrients [19]. The objectives of the “Mid-Day-Meals Scheme” are increasing school participation, preventing classroom hunger, facilitating healthy growth of children, using the opportunity to inculcate good habits such as hand washing, fostering social equality, enhancing gender equality, and offering psychological benefits [20].

A report evaluating the success of this program stated that for a cost as low as three cents per child per school day, it reduced the protein deficiency by 100%, calorie deficiency by 30%, and Iron deficiency by 10% and also reduced hunger and Protein-Energy Malnutrition [18].

## 5. “Pulse Polio” Campaign

Before the launch of the Global Polio Eradication Initiative by in the World Health Assembly (WHA) 1988, polio crippled an estimated 200,000 children in India each year. Despite launching the polio eradication initiative in 1995, as recently as in 2009, India reported close to half the cases reported globally. However, in 2012 India achieved a milestone of passing one full year without recording any polio case; as a result India has been removed from the list of polio endemic countries. The success story was written by millions of frontline workers—vaccinators, social mobilizers, community workers, health workers, religious leaders, influencers, and parents—in often difficult circumstances and environments [21].

The pulse polio has been upheld as an immensely successful public health campaign, publicised widely and implemented thoroughly. There exists an infrastructure of local community health workers (Anganwadi workers) who work under the district and Taluka health and education authorities. The campaign also made use of the wide network of primary health care centers throughout the country for the successful monitoring and control of the campaign [21].

The lesson learnt from the above-mentioned two public health campaigns is that for a national health promotion program to become successful, a combination of planning at the top level and implementation at the local level is imperative.

Although there has not been an example of an effective oral health promotion program integrated with the overall health promotion in India, a program that has caught attention in the recent times is the “Fit for School” program that is currently underway in Philippines [22, 23]. This program can be considered as a model program and replicated in the rural Indian scenario. Described below are the important aspects of the program.

## 6. “Fit for School” Program

A school health program in Philippines currently in place for 630,000 children (2010) and targeting six million children in the next three years is based on the partnership between the Department of Education (Ministry) and local governments with the support of German Development Corporation and GlaxoSmithKline [22, 23].

The program has three essential components: hand washing, tooth brushing with a fluoride toothpaste (i.e., specially made available), and periodic deworming. It involves the participation of Parent-Teacher Community Association (PTCA: a prerequisite for implementing the program), teachers (in a supervisory role), the school principals (to ensure that the activity takes place and the consumables are available and to communicate with the school nurse and the PTCA), and a school nurse (for monitoring twice in a year). The costs for one toothbrush, 60 mL toothpaste, soap, and two, deworming tablets are 0.5 Euros per year [22].

## 7. Potential for Infant and Child Oral Health Promotion

The literature supports the promotion of hand washing in developing countries as a cost-effective measure in reducing diarrhoeal deaths [24]. Furthermore, hand washing alone is reported to lower the respiratory infections by 16% [25].

The literature also reports that a key to success of a health program is the participation of stakeholders in it. The partnerships of parent teacher associations in schools, local professional groups such as the dental bodies, social organizations, and public health schools are essential for the successful implementation of an oral health program [26].

Schools are an effective platform for the promotion of oral health because they cover a significantly large population (one billion) across the world [27]. The concept of Health Promoting Schools of the World Health Organization has met success through implementation in schools and influenced knowledge and behaviours of children [28]. A holistic approach for child health in India based on the health promotion in preschools and schools for children between 3 and 16 years of age can be conceptualized to integrate oral and general health of Indian school children particularly in the deprived communities.

There has not been a national oral health program for the rural (as well as the urban) India till date. Hence, as mentioned earlier, one has to look for an evidence of other successful public health campaigns in recent times to understand how to develop a model in terms of developing policies, making use of existing public health plans, using the currently available manpower and resources and promoting it well across the country crossing most barriers. An oral health promotion program can have three components: health education, fluoride use, and nutrition. 

Education of community health workers using appropriate tools can be taken up as a step in the development of the infant and child oral health promotion program. Utilization of existing machinery that made successful the pulse polio campaign in India for the infant and child oral health promotion can be a pragmatic strategy. Already, initiatives have been taken for using the machinery towards routine immunization coverage of children. Adding an oral health component to early childhood health promotion can be a practical approach. A tool developed at the University of Toronto, a DVD-video containing evidence-based information about infant oral health care and prevention containing comprehensive anticipatory guidance in the areas of pregnancy, oral development, teething, diet and nutrition, oral hygiene, fluoride use, acquisition of oral bacteria, feeding and oral habits, causes and consequences of early childhood caries, trauma prevention, early dental visits, and regular dental visits, can be adapted with necessary translation, modification, and validation for this purpose [29]. Also, print material in the local languages can be developed for the purpose. Fluoridated toothpastes and fluoride varnishes have been proved to be effective in caries reduction [30]. For the manpower required to train the community health workers and fluoride varnish applications and initiating the preschool and school tooth brushing programs, dental colleges across the country can participate. There exist close to 300 dental colleges in India. In the recently upgraded curriculum of dentistry by the Dental Council of India, emphasis is given on public health dentistry (wherein the interns need to be engaged in community programs for a period of three months). Each college has between 40 and 100 interns in each academic year; all together comprising a sizable manpower that can be utilized for the task. Also, most dental colleges have satellite dental centers and mobile dental vans for community outreach programs which can be utilized, too. The academic staff of the dental colleges can avail of the research opportunities through the program and be helpful in monitoring the implementation and evaluation. The members of local branches of Indian Dental Association can be helpful in the propagation of the initiative.

Proposed below is a model for the infant and child oral health promotion in rural India based on the considerations discussed above.

## 8. Model for Infant and Child Oral Health Promotion in Rural India

The model for the Infant and Child Oral Health Promotion in Rural India can include activities specific to age groups. The program for children up to age 6 years can be divided into three age-categories based on the age groups.

### 8.1. Age Group 0–2 Years

The community health workers in India are in continuous contact with the families for routine immunizations of children and provision of healthcare and government aid. An upstream approach to educate these workers (using audiovisual tools) so that the information reaches the masses (verbally as well as in print) can be initiated. These community health workers also have access to the health records of all children, which can be valuable. 

### 8.2. Age Group 2-3 Years

Most children would have a complete primary dentition during this period and thus are in the best position to benefit from 6 monthly fluoride varnish applications (twice during the year). The community health workers can assist parents bring the children to Anganwadi branches or the primary healthcare centers for fluoride varnish application programs. The fluoride varnish applications can be carried out in these settings or a mobile dental van by engaging the interns of nearby dental schools. A voluntary support of the local branch of the Indian Dental Association is also desirable.

### 8.3. Age Group 3–6 Years and 6–16 Years

The preschool and school programs can involve a combination of previously discussed programs “The Mid-Day-Meals Scheme” that runs mandatorily in all the public schools across the country and two components (hand-washing and tooth brushing) of the “Fit for School” program that is underway in Philippines. 

The details of the program are summarized in Table 1.

Recommendations of the proposed model are based on the five principles of the Ottawa Charter, which are relevant even 25 years after their first appearance [31].

The program demands “building public policies” such as a directive from a local government (from the Taluka Panchayat or a district authority). It further needs “creation of supportive environments” for the purchase of consumables at subsidized rates (by means of partnerships with industry) and to overcome practical barriers (e.g., cultural). It calls for “strengthening community action” by the active participation of the stakeholders (for instance, an active role of the parents in the parent teacher association). Moreover, it seeks to “develop personal skills” of the community health workers, local dentists in terms of leadership and teaching, which can help sustain the program in a self-reliant manner. It also asks for “reorientation of health services” only to a minimum, only for the supervisory and quality control of the intervention through periodic health monitoring, audits, and so forth.

## 9. Monitoring and Evaluation

Health programs are complex and often dynamic rather than static. “A health program is an interaction of health concerns, program elements and its positive and negative drivers” [32]. The planning, monitoring, and evaluation of the proposed program must take into consideration the steps outlined in the “planning cycle,” such as identifying needs, assessing resources, determining priorities, setting goals and objectives, implementing the program, and evaluating the program [26]. 

Success of the model discussed above can be evaluated on the basis of both the process evaluation and clinical outcome evaluation. For the purpose of monitoring, a team of experts (academicians in Public Health Dentistry from dental colleges) and local members of Indian Dental Association can be built.

The success of the proposed program will depend on the achievements in the measures of oral and systematic health. This program provides an opportunity to assess the following outcome measures.

A reduction in caries increment and improvement in oral health status can be considered as the long term oral health measures. Reduction in diarrhoeal and respiratory infections and improvement in the nutritional status of the children can be regarded as the systemic health measures. Also, whether the reduced hunger, improved nutrition, and health have any influence on the school attendance and performance of children can be evaluated. The improvement in the knowledge, attitude, and behaviours of the children and their families can also be the measures of interest. In practical terms, the cost-benefit analysis and viability of the program need to be considered among the other measures. 

It could be possible to include a research component with the following outcome measures for the oral health promotion evaluation.

- indexes: dmft/DMFT, SiC, pufa/PUFA, Early Childhood Oral Health Impact Scale (ECOHIS) for 5- and 12-year-old children (WHO index ages),
- assessment of knowledge and knowledge retention in Anganwadi workers, school teachers, and parents (using prevalidated questionnaires),
- acceptance to and sustainability of fluoride varnish program in 2-3 year-old children (through qualitative assessment),
- acceptance to and sustainability of the program of preschool and school tooth brushing with fluoride toothpaste in 3–16-year-old children (through qualitative assessment),
- comparison of dental attendance pre- and postprogram (based on health records).

## 10. Limitations

The structure of the program demands participation of central, state, and local governments to develop policies and give directives. In a country like India, this can be a challenge due to the sheer size of and diversities within the country and priorities before the government/s and differences in political wills in different regions. There also exist cultural and social differences, which may pose challenges. Shortage of water, particularly in draught affected areas, can be a problem for setting up hand washing and tooth brushing stations that need water supply. 

The program is aimed at improving health of the children in the deprived communities. The real shortcoming of the program could be that it may not be sufficient to tackle the causes of the ill health of the children beyond the school settings. Whether it extends the benefits of the practices of hygiene beyond the boundaries of school, whether the improper sanitation and contaminated drinking water supplies continue to be the reasons for poor health statuses of children, and whether these reasons will in turn mask the success of the program will be the pragmatic challenges for the program. Furthermore, the rural India is deprived of government funded dental services, which means that people would not be able to seek dental care in spite of increased awareness and need. Moreover, propagation and monitoring the program needs building a network of leaders. Lack of experience and incentives to run the program may affect the initiation and sustenance of the program.

Public-private partnership could be a way to overcome the challenges anticipated in the implementation of the model. A need for encouraging the pharmaceutical industry and the social sector to take greater responsibility to support the public health system and health research in India has been identified [33]. The same will be necessary to bring this model to reality in terms of its potential scope and magnitude.

## 11. Conclusion

### 11.1. “Think Globally, Act Locally”

Large population, diversities within the country, staggering high malnutrition and infectious diseases in children, growing concern of noncommunicable diseases and dental diseases, and poor investments in health are the considerations while developing a model for oral health promotion in infants and children. The model discussed here is a practical, cost-effective, evidence-based, directed population approach for oral health promotion in infants and children, aiming at overall health promotion (an integrated approach) suggesting the use of the existing machinery of the recently successful public health campaigns in India and the manpower of the widespread network of dental colleges and branches of Indian Dental Association. Although it can be challenging to bring it to reality, the model perhaps has a potential to bridge the gap between the enormous need of and miniscule effort in the oral health promotion targeting infants and children.

## Figures and Tables

**Table 1:** Model for infant and child oral health promotion agewise initiatives.

Age group	Settings	People to engage with	Scope, components and tools
0–2 years	Primary healthcare centers, Anganwadi branches	Anganwadi workers (direct contact), parents (indirect contact)	Oral Health Education: for Anganwadi workers using a DVD on infant oral care and for parents using printed booklets
2-3 years	Primary healthcare centers, Anganwadi branches	Anganwadi workers and parents (direct contact)	Oral Health Education: for parents using printed booklets Fluoride varnish program in mobile dental van/or dental satellite centers
3–6 years	Preschools	Preschool teachers and parents (Parent-Teacher Associations)	Tooth-brushing program with fluoride toothpaste in combination with the Mid-Day Meals Scheme and hand washing
6–16 years	Schools	School teachers and parents (Parent-Teacher Associations)	Tooth-brushing program with fluoride toothpaste in combination with the Mid-Day Meals Scheme and hand washing
